# Anti-inflammatory effect of cholera toxin B subunit in experimental stroke

**DOI:** 10.1186/s12974-016-0610-y

**Published:** 2016-06-13

**Authors:** Lei Zhang, Yanxia Huang, Yinyao Lin, Yilong Shan, Sha Tan, Wei Cai, Haiyan Li, Bingjun Zhang, Xuejiao Men, Zhengqi Lu

**Affiliations:** Department of Neurology, The Fifth Affiliated Hospital of Sun Yat-sen University, No. 52 Meihuadong Road, Zhuhai City, China; Department of Neurology, The Third Affiliated Hospital of Sun Yat-sen University, No. 600 Tianhe Road, Guangzhou City, China

**Keywords:** Cholera toxin B subunit, Stroke, Inflammation

## Abstract

**Background:**

Cholera toxin B subunit (CTB) has multifaceted immunoregulatory functions. Immunity plays an important role in the mechanism of stroke. However, little is known about whether CTB is beneficial for stroke.

**Methods:**

CTB was administered intraperitoneally after ischemia to rats subjected to transient focal ischemia. Infarct volumes, body weight loss, and neurologic deficits were measured. Cytokines, microglia/macrophage activation, and transcriptional factors in the ischemic brain were tested. The mRNA expressions of IL-1β and TNF-α were tested in the microglia/macrophage isolated from the ischemic hemisphere. γδT cells, IL-17-producing γδT cells, Th17 cells, and regulatory T (Treg) cells in the ischemic brain were tested. γδT cells and Treg cells in the peripheral blood were also evaluated.

**Results:**

CTB reduced infarct volumes, neurologic deficits, and body weight loss after ischemia. At 24 h after ischemia, CTB downregulated the levels of IL-1β, TNF-α, NF-kB p65, phosphorylated-ERK1/2, and microglia/macrophage activation and suppressed NF-kB binding activity, but did not affect the level of ERK1/2. The mRNA expressions of IL-1β and TNF-α in the microglia/macrophage isolated from the ischemic hemisphere were suppressed after CTB therapy. In the ischemic hemisphere, CTB treatment reduced the levels of γδT cells, IL-17-producing γδT cells, and IL-17 at both 24 and 72 h after ischemia, while Th17 cells were not affected. After CTB treatment, the levels of Treg cells, TGF-β, and IL-10 remained unchanged at 24 h and upregulated at 72 h after ischemia. Inactivation of Treg cells using anti-CD25 attenuated the increase of TGF-β and IL-10 induced by CTB at 72 h after ischemia. In the peripheral blood, CTB increased Treg cells and suppressed γδT cells at 24 h after ischemia. And then at 72 h after ischemia, it increased Treg cells but did not impact γδT cells. CTB had no effect on cytokines, transcription factors, infiltrating γδT cells, and Treg cells in the brain of shams. In the peripheral blood of shams, CTB increased Treg cells at both 24 and 72 h, while it did not affect γδT cells.

**Conclusions:**

CTB decreased neurologic impairment and tissue injury after cerebral ischemia via its immunomodulatory functions, including inhibiting microglia/macrophage activation, suppressing γδT cells, and inducing production of Treg cells, thus regulating the secretion of related cytokines. Suppression of NF-kB and ERK1/2 pathways is involved in the neuroprotective mechanism of CTB.

## Background

Ischemic stroke is a leading cause of disability and death in the world [1]. Inflammation plays an important role in the pathogenesis of ischemic stroke. Many anti-inflammatory materials have proven successful in animal models [2, 3], but attempts to translate them into clinical application have been unsuccessful [4, 5]. So the search for suitable anti-inflammatory substances is still essential.

Cholera toxin is composed of one A subunit and five B subunits. The cholera toxin B subunit (CTB) is devoid of toxicity and capable of immunomodulation. Researchers found that CTB could act as immunomodulatory molecule capable of suppressing Th1-associated immune responses [6]. Therefore, CTB began to be studied as a therapy for T cell-mediated autoimmune diseases in animal models. Studies showed that coupling myelin basic protein or insulin to CTB could be used as effective therapies against experimental allergic encephalomyelitis in the rats and diabetes in the nonobese diabetic (NOD) mice, respectively [7, 8]. Besides, CTB used alone could prevent the development of diabetes in NOD mice [9]. Studies have demonstrated that CTB can inhibit the activation of macrophage induced by lipopolysaccharide [10] and induce the production of regulatory T (Treg) cells [11]. Suppressing Th1-associated immune responses, enhancing Th2-associated immune responses, inhibiting the activation of macrophage/microglia, and inducing Treg cells are protective in stroke [12–14]. However, little is known about whether CTB is beneficial for stroke. In the present study, we investigated whether CTB had a neuroprotective effect in the rat model of middle cerebral artery occlusion (MCAO) and the potential mechanisms for its neuroprotection.

## Methods

### Animals

Male Sprague–Dawley rats weighing 250–300 g (Laboratory Animal Center of Sun Yat-sen University, Guangzhou, China) were housed in groups of four animals per cage under controlled temperature (24.0 ± 2 °C) and a 12-h light/dark cycle, with food and water available. The protocol was carried out according to the National Institute of Health Guide for the Care and Use of Laboratory Animals. The animals were randomly divided into four groups (*n* = 8 for each group): MCAO group, low dose group (animals with MCAO and 0.4 mg/kg CTB treatment), high dose group (animals with MCAO and 0.8 mg/kg CTB treatment), CTB-sham group (animals with 0.8 mg/kg CTB treatment), and sham operated group. Because separate groups of animals were required for different tests and time points, a total of 320 rats were used. In detail, nine flocks of rats were used, one flock for TTC staining, two flocks for the flow cytometry studies at 24 and 72 h, respectively, two flocks for the qPCR, ELISA, and immunohistochemistry at 24 and 72 h, respectively, two flocks for microglia isolation from the ischemic hemisphere at 24 and 72 h, respectively, and two flocks for inactivation of Treg cells by anti-CD25 at 24 and 72 h, respectively. Considering that the neurodeficit scores at 72 h were unavailable in three flocks because of sacrifice at 24 h and inactivation of Treg cells could deteriorate ischemic stroke, we presented the neurodeficit scores and body weights at both 24 and 72 h of four flocks. For the same reason, when analyzing mortality rate, the two flocks for inactivation of Treg cells were not included.

Blinding was used at all stages throughout the study, including neurodeficit scoring and sample and tissue analysis.

### Middle cerebral artery occlusion model

The food was withdrawn 12 h before the surgical procedure. The right MCAO was performed using an intraluminal filament model [15]. Briefly, the rats were anesthetized with an intraperitoneal injection of 3 % amobarbital (13 ml/kg). The right common carotid artery was exposed through a ventral midline neck incision. A monofilament (SUNBIO, China) was introduced into the external carotid artery and advanced into the circle of Willis via the internal carotid artery to occlude the origin of the right middle cerebral artery. The filament was inserted 18–20 mm from the carotid bifurcation, confirmed with mild resistance. Successful occlusion was confirmed by an 80–90 % reduction in cerebral blood flow measured by laser Doppler flowmetry. Ninety minutes after the induction of ischemia, the rat was re-anesthetized to allow the withdrawal of the filament and reperfusion was verified by laser Doppler flowmetry. Then, the animals were returned to their cages and given free access to food and water. CTB-sham group and sham group rats went through all surgical procedures except the monofilament insertion. During the surgery, rectal temperature was controlled at 37.0 °C with a heating pad. Body weights were measured before surgery and at 24 and 72 h after MCAO. Inclusion criteria were (1) successful occlusion and reperfusion and (2) neurologic deficit score ≥1. Exclusion criteria were (1) premature death and (2) cerebral hemorrhage or subarachnoidal hemorrhage.

### Drug administration

CTB (Sigma) was dissolved in saline to prepare concentrations of 0.5 mg/ml. For low dose group and high dose group, CTB at doses of 0.4 and 0.8 mg/kg (added with saline to a total volume of 1 ml) was administered respectively by intraperitoneal injection, immediately after cerebral ischemia and every 24 h for 3 days. For CTB-sham group, CTB at doses of 0.8 mg/kg (added with saline to a total volume of 1 ml) was administered by intraperitoneal injection, immediately after cerebral ischemia and every 24 h for 3 days. We used high dose CTB in CTB-sham group to make sure that the effect of CTB treatment on baseline results of our study could be sufficiently demonstrated. In the case of the MCAO and sham operated group, equal volume saline was administered in the same manner. To inactivate Treg cells, 300 μg of anti-CD25 was injected intraperitoneally 48 h before ischemia induction.

### Measurement of infarct volume

At 72 h after MCAO, the rats were sacrificed under deep anesthesia with an overdose of pentobarbital (*n* = 8). To quantify ischemic damage, brains were dissected and cut into six coronal slices of 2-mm thickness, incubated in a 2 % solution of triphenyl tetrazolium chloride (TTC, Sigma) at 37 °C for 15 min and immersion-fixed in a 4 % paraformaldehyde. TTC-stained sections were photographed, and the digital images were analyzed using Image-Pro Plus 5.1. The lesion volume was calculated by multiplying the area by the thickness of slices. To compensate for the effect of brain edema, percentage hemisphere lesion volume (%HLV) was calculated by the following equations [16]: % HLV = {[total infarct volume ‐ (right hemisphere volume ‐ left hemisphere volume)] / left hemisphere volume} × 100 %.

### Analysis of neurologic deficit scores

Neurological function was evaluated at 24 and 72 h after MCAO. The deficits were scored on a modified scoring system based on that developed by Longa et al. [15] as follows: 0, no deficits; 1, difficulty in fully extending the contralateral forelimb; 2, unable to extend the contralateral forelimb; 3, mild circling to the contralateral side; 4, severe circling; and 5, falling to the contralateral side.

### Western blotting

For Western blotting analysis, the rats were sacrificed immediately 24 h after MCAO. The cytosolic and nuclear extracts from the ipsilateral cerebrum were prepared according to the manufacturer’s instructions (Thermo). Briefly, the tissue was homogenized in five volumes of homogenization buffer A [20 mM *N*-2-hydroxyethylpiperazine-*N*′-2′-ethanesulfonic acid (HEPES), 1.5 mM MgCl_2_, 10 mM KCl, 1 mM EDTA, 1 mM ethylene glycol-bis(2-aminoethyl ether) tetraacetic acid (EGTA), 250 mM sucrose, 0.1 mM phenylmethanesulfonyl fluoride (PMSF), 1 mM dithiothreitol (DTT), and 10 μg/ml of each of aprotinin, pepstatin A, and leupeptin, pH 7.9]. The sample was centrifuged at 750*g* at 4 °C for 15 min to separate the sample into supernatant A and pellet A. Pellet A, containing the nuclear fraction, was resuspended in 90 μl of buffer B (20 mM HEPES, 1.5 mM MgCl_2_, 20 mM KCl, 0.2 mM EDTA, 0.5 mM EGTA, 0.2 mM PMSF, 0.5 mM DTT, and 10 μg/ml of each of aprotinin, pepstatin A, and leupeptin, pH 7.9) and mixed with 30 μl of buffer C (20 mM HEPES, 1.2 mM KCl, 0.2 mM EDTA, 0.2 mM PMSF, 0.5 mM DTT, and 10 μg/ml of each of aprotinin, pepstatin A, and leupeptin, pH 7.9). The sample was placed on ice for 30 min during the extraction and then centrifuged at 12,000*g* for 30 min at 4 °C. The supernatant containing the nuclear fraction was transferred and stored at −70 °C. Supernatant A, containing the cytosolic protein, was further centrifuged at 16,000*g* for 30 min at 4 °C to separate supernatant B from pellet B. Supernatant B was used as the cytosolic fraction and pellet B was discarded. Nuclear pellet purity was assured by microscopic observation. To exclude cross-contamination between the nuclear and cytosolic fractions, specific antibodies of Lamin A (Santa Cruz Biotechnology) and NF-kB (p50/p105) (eBioscience) were used as individual markers for each fraction. The Lamin A protein served as nuclear contamination marker and the p105, the precursor of NF-kB p50, served as a cytoplasmic contamination marker. The protein concentrations were determined using a BCA protein assay reagent kit (Novagen). As much as 30 μg of protein was separated by SDS/PAGE, transferred 2 h to PVDF membranes, the nonspecific binding of antibodies was blocked with 5 % nonfat dried milk in PBS and then incubated with the primary antibodies at 4 °C overnight: a rabbit anti-rat NF-kB p65 antibody (1:2000, Millipore), a rabbit anti-rat p-ERK1/2 antibody (1:1000, Cell Signaling Technology), a rabbit anti-rat β-actin (1:200, Millipore), and a rabbit anti-rat histone H3 (1:100, Millipore). After three washes with PBS containing 0.1 % Tween-20, the second antibodies were incubated with membranes for 1 h at room temperature. The relative density of bands was analyzed using an imaging densitometer (LI-COR Bioscience). β-actin and histone H3 were used as an internal control for the cytosolic and nuclear fractions, respectively.

### EMSA

To determine the NF-kB binding activity in the ischemic hemisphere, electrophoretic mobility shift assay (EMSA) was performed. The oligonucleotides containing NF-kB binding site (5′-AGTTGAGGGGACTTTCCCAGGC-3′) and AP-1 binding site (5′-CGCTTGATGAGTCAGCCGGAA-3′) were used as DNA probes (Promega). The DNA probes were radio-labeled with [γ-32p] ATP using the T4 kinase according to the manufacturer’s instructions. For binding reaction, the radio-labeled probes were incubated with 5 μg of nuclear extracts. The binding buffer contained 10 mM Tris–HCl (pH 7.5), 50 mM NaCl, 0.5 mM EDTA, 1 mM DTT, 1 mM MgCl_2_, 4 % glycerol, and 2 μg poly (dI-dC). The reaction mixtures were left at room temperature to precede binding reaction 20 min. For supershift assays, antibody to NF-kB was pre-incubated with nuclear extracts for 30 min at 4 °C before the addition of radio-labeled probes. The final reaction mixtures were analyzed in a 6 % nondenaturing polyacrylamide gel with 0.25× Tris-borate/EDTA as an electrophoresis buffer. The specificity of binding was also examined by competition assay with a 100-fold excess of nonlabeled NF-kB probes. Nonspecific band was identified by changing the order of addition, since the order of addition could influence nonspecific binding in gel shift assays. The figure was presented with minimum effect of nonspecific band.

### Isolation of microglia/macrophage from the ischemic hemisphere

Isolation of microglia/macrophage with preserved phenotypes was described in a previous study [17]. Briefly, after perfusion with ice-cold PBS, the ischemic hemisphere was dissected and enzymatically digested using Neural Tissue Dissociation Kit (Miltenyi Biotec) for 35 min at 37 °C. Further processing was performed at 4 °C. Tissue debris was removed by passing the cell suspension through a 40-μm cell strainer. Cells were resuspended in 30 % Percoll (GE Healthcare) and centrifuged for 10 min at 700*g*. The supernatant containing the myelin was removed, and the pelleted cells were washed with HBSS. Then, cells were stained with PE-conjugated anti-CD11b antibodies (Miltenyi Biotec) in IMAG buffer (PBS supplemented with 0.5 % BSA and 2 mM EDTA) for 10 min followed by incubation for 15 min with anti-PE magnetic beads. CD11b+ cells were separated in a magnetic field using MS columns (Miltenyi Biotec). The amounts of antibodies and magnetic beads were calculated based on the number of cells obtained after myelin removal, according to the manufacturer’s guidelines.

### Cytokine enzyme-linked immunosorbent assay

Ischemic brain tissue were collected and frozen at −80 °C immediately until analysis of cytokine protein concentrations with commercial kits for the quantitative assay of TNF-α, IL-1β, IL-17, IL-10, and TGF-β (RayBio). Individual ELISA was used for individual cytokines. For the active form of TGF-β measurement, recombinant latent TGF-β (1 μg/300 μl) was incubated with sTSP (3 μg/300 μl) for 5 min at 37 °C in a total volume of 300 μl of PBS in siliconized microcentrifuge tubes. The samples were then tested for active TGF-β using a TGF-β ELISA Predicta kit. The ELISA is selective for active TGF-β and only minimally recognizes latent TGF-β.

### RNA isolation and quantitative real-time PCR

To evaluate the cytokines and signaling molecules in both transcriptive and translative levels, we conducted real-time PCR to test the mRNA expressions after measuring protein cytokine levels, so that the data were more sufficient and convincing. The ischemic brain tissue prepared by Percoll gradient centrifugation and the microglia/macrophage isolated from the ischemic hemisphere were lysed in RNAiso (Takara), respectively. Real-time PCR was performed on cDNA samples using SYBR Green qPCR SuperMix (Invitrogen). The primer sequences used in this study were as follows: TNF-α (primers, sense 5′-TGA AGT AGT GGC CTG GAT TGC-3′; antisense 5′-GAC ATT CCG GGA TCC AGT GA-3′), IL-1β (primers, sense 5′-GTG CTG TCT GAC CCA TGT GA-3′; antisense 5′-CAC AGG GAT TTT GTC GTT GCT-3′), IL-17 (primers, sense 5′-CTC TGC AAA CTT CCT TGT CT-3′; antisense 5′-GAA GGC AGA CAA TTC TAA CC-3′), IL-10 (primers, sense 5′-TCT ACA AGG CCA TGA ATG AG-3′; antisense 5′-GAG AGA GGT ACA AAC GAG G-3′), TGF-β1 (primers, sense 5′-TGC TTC AGC TCC ACA GAG AA-3′; antisense 5′-TGG TTG TAG AGG GCA AGG AC-3′), NF-kB p65 (primers, sense 5′-GAC GAT CTG TTT CCC CTC AT-3′; antisense 5′-GCT TCT CTC CCC AGG AAT AC-3′), ERK1 (primers, sense 5′-GGA CCG GAT GTT AAC CTT TA-3′; antisense 5′-TGG TTC ATC TGT CGG ATC AT-3′), ERK2 (primers, sense 5′-CGT ACC TGG AGC AGT ATT ATG A-3′; antisense 5′-CCA GCT CCA TGT CAA ACT TG-3′), β-actin as an internal standard (primers, sense 5′-AGG GAA ATC GTG CGT GAC AT-3′; antisense 5′-GAA CCG CTC ATT GCC GAT AG-3′). The reactions were initially heated at 50 °C for 2 min; then at 95 °C for 2 min, 95 °C for 15 s, and 60 °C for 32 s, total of 40 cycles; and finally stopped at 60 °C for 5 min. The relative gene expression was calculated using the ΔΔCt method, the samples were normalized to the expression of β-actin, and the untreated sample was used as a calibrator.

### Immunohistochemistry

Microglia activation was assessed by staining for Iba1 detected by immunohistochemical analysis. Rats were deeply anesthetized at different time points and then were perfused transcardially with normal saline followed by ice-cold 4 % paraformaldehyde in phosphate-buffered saline. Brain tissues were removed and fixed overnight in 4 % paraformaldehyde at 4 °C and then immersed in 30 % sucrose. They were cut to 2-mm-thick coronal sections and embedded in paraffin and further sliced at a thickness of 5 μm. The slides were deparaffinized, sequentially rehydrated in graded alcohol, and then immersed in PBS (pH 7.4). Slides were then microwaved for 2 min in antigen unmasking solution, cooled, and washed three times for 2 min in PBS. Sections were immersed for 25 min in 3 % hydrogen peroxide in distilled water to eliminate endogenous peroxidase activity, then blocked in immunohistochemical grade 1 % bovine serum albumin in PBS for 1 h and diluted goat serum for 30 min to reduce nonspecific staining. Sections were incubated overnight with an anti-rat monoclonal antibody against Iba1 (Abcam). Peroxidase-conjugated anti-rabbit IgG (BD Biosciences) was used as the secondary antibody (incubating for 30 min at 37 °C). Antibodies were detected using the DAB kit (Boster, Wuhan, China) following the manufacturer’s instructions. Finally, the slides were observed using a light microscope. In specimens, the positive cells were counted in six selected areas of ischemic border and the corresponding areas of contralateral hemispheres for each case and expressed as the number of immunopositive cells/mm^2^.

### Flow cytometry

The ischemic hemisphere and blood samples at various time points after MCAO were collected for flow cytometric analysis of leukocyte subpopulations. Individual cell suspensions from the ischemic hemisphere and peripheral mononuclear blood cells (PBMC) were prepared. Rat antibodies were as follows (all from eBioscience): FITC-labeled anti-rat CD4 antibodies, PE-labeled anti-rat CD25 antibodies, PE-labeled anti-rat Foxp3 antibodies, APC-labeled anti-rat CD3 antibodies, PE-labeled anti-rat IL-17A antibodies, and FITC-labeled anti-rat gamma delta TCR antibodies. Surface staining was performed for 15 min with the corresponding mixture of fluorescently labeled antibodies. After staining of surface markers, cells were fixed, permeabilized using fixation buffer in conjunction with permeabilization buffer (eBioscience) and stained with antibodies anti-cytokines and the corresponding isotype controls. For detection of γδT cells, IL-17-producing γδT cells, and Th17 cells, individual aliquots of 1 × 10^6^ T cells were stimulated with phorbol 12 myristate 13-acetate (PMA) (50 ng/ml; Sigma) and ionomycin (1 μg/ml; Sigma) and in the presence of monencin (1.7 μg/ml; Sigma) at 37 °C and 5 % CO_2_ for 4 h before they were collected and stained with antibodies. FACS analysis was performed on a BD FACSAria and analyzed using FlowJo software.

### Statistical analysis

All data in the text, tables, and figures are given as the mean ± SD. Data were analyzed by a two-tailed *t* test or one-way analysis of variance (ANOVA) followed by the post hoc Student–Newman–Keuls (S–N–K) *t* test for multiple comparisons. Qualitative data were analyzed with the *χ*^2^ test, Differences were considered statistically significant with *p* < 0.05. Statistical analyzes were carried out using SPSS 17.0.

## Results and discussion

### CTB ameliorated neurological dysfunctions, mortality rate, and body weight loss

**Fig. 1:**
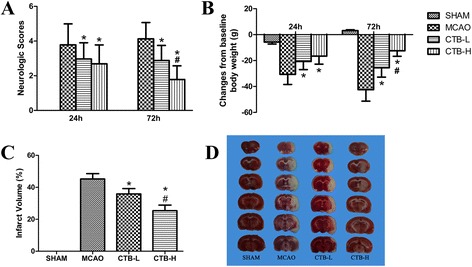
Effect of CTB on neurological deficit scores, body weight, and infarct volume after MCAO. **a** Elevated neurological deficit scores after MCAO were significantly attenuated by CTB, and neurologic deficits were significantly ameliorated in the CTB-H group compared with the CTB-L group, both at 24 and 72 h. **b** CTB ameliorated body weight loss at 24 h after cerebral ischemia, while no significant difference was found between the CTB-L group and CTB-H group. At 72 h after cerebral ischemia, body weight loss was significantly ameliorated by CTB, and body weight loss in the CTB-H group was significantly less than that in the CTB-L group. **c** Quantification of infarct volume at 72 h. The ratio of corrected infarct volume to the nonischemic hemisphere volume was calculated for the cerebral infarct size. Infarct volume was decreased at 72 h with CTB treatment and infarct volume was significantly reduced in the CTB-H group compared with the CTB-L group. **d** Representative TTC stained brain sections of different groups were shown. *Bars* represent mean ± SD (*n* = 8); *p < 0.05 vs MCAO group, and #*p* < 0.05 vs CTB-L group

Neurologic deficits, evaluated at 24 h after cerebral ischemia, were significantly reduced in low dose CTB (CTB-L) group and high dose CTB (CTB-H) group compared with MCAO group, respectively (CTB-L vs MCAO, *p* = 0.001; CTB-H vs MCAO, *p* < 0.001; Fig. 1a). And neurologic deficits were not significantly different in the CTB-H group compared with the CTB-L group (Fig. 1a). At 72 h after cerebral ischemia, neurologic deficits were significantly reduced in the CTB-L group and the CTB-H group compared with the MCAO group, respectively (CTB-L vs MCAO, *p* < 0.001; CTB-H vs MCAO, *p* < 0.001; Fig. 1a). And neurologic deficits were significantly ameliorated in the CTB-H group compared with the CTB-L group (*p* < 0.001; Fig. 1a). No rats appeared with neurological impairment symptoms in the sham group.

No rats died in the sham group. The mortality rates of the MCAO group, CTB-L group, and CTB-H group were 22.2 % (16/72), 13.8 % (9/65), and 6.7 % (4/60), respectively, with significant difference (*p* = 0.041).

Similar with neurologic deficits, body weight loss at 24 h after cerebral ischemia was significantly ameliorated in the CTB-L group and CTB-H group compared with the MCAO group, respectively (CTB-L vs MCAO, *p* < 0.001; CTB-H vs MCAO, *p* < 0.001; Fig. 1b), while no significant difference was found between the CTB-L group and CTB-H group (Fig. 1b). At 72 h after cerebral ischemia, body weight loss was significantly ameliorated in the CTB-L group and CTB-H group compared with the MCAO group, respectively (CTB-L vs MCAO, *p* < 0.001; CTB-H vs MCAO, *p* < 0.001; Fig. 1b). And body weight loss in the CTB-H group was significantly less than that in the CTB-L group (*p* < 0.001; Fig. 1b).

### CTB reduced brain infarction

Infarct volume was measured by TTC at 72 h after cerebral ischemia. Infarct volumes in both the CTB-L group and CTB-H group were significantly reduced compared with the MCAO group, respectively (CTB-L vs MCAO, *p* < 0.001; CTB-H vs MCAO, *p* < 0.001; Fig. 1c, d). And infarct volume in the CTB-H group was significantly smaller than that in the CTB-L group (*p* < 0.001, Fig. 1c, d).

### CTB downregulated pro-inflammatory cytokines in the ischemic brain and microglia/macrophage isolated from the ischemic hemisphere and upregulated anti-inflammatory cytokine in the ischemic brain

**Fig. 2:**
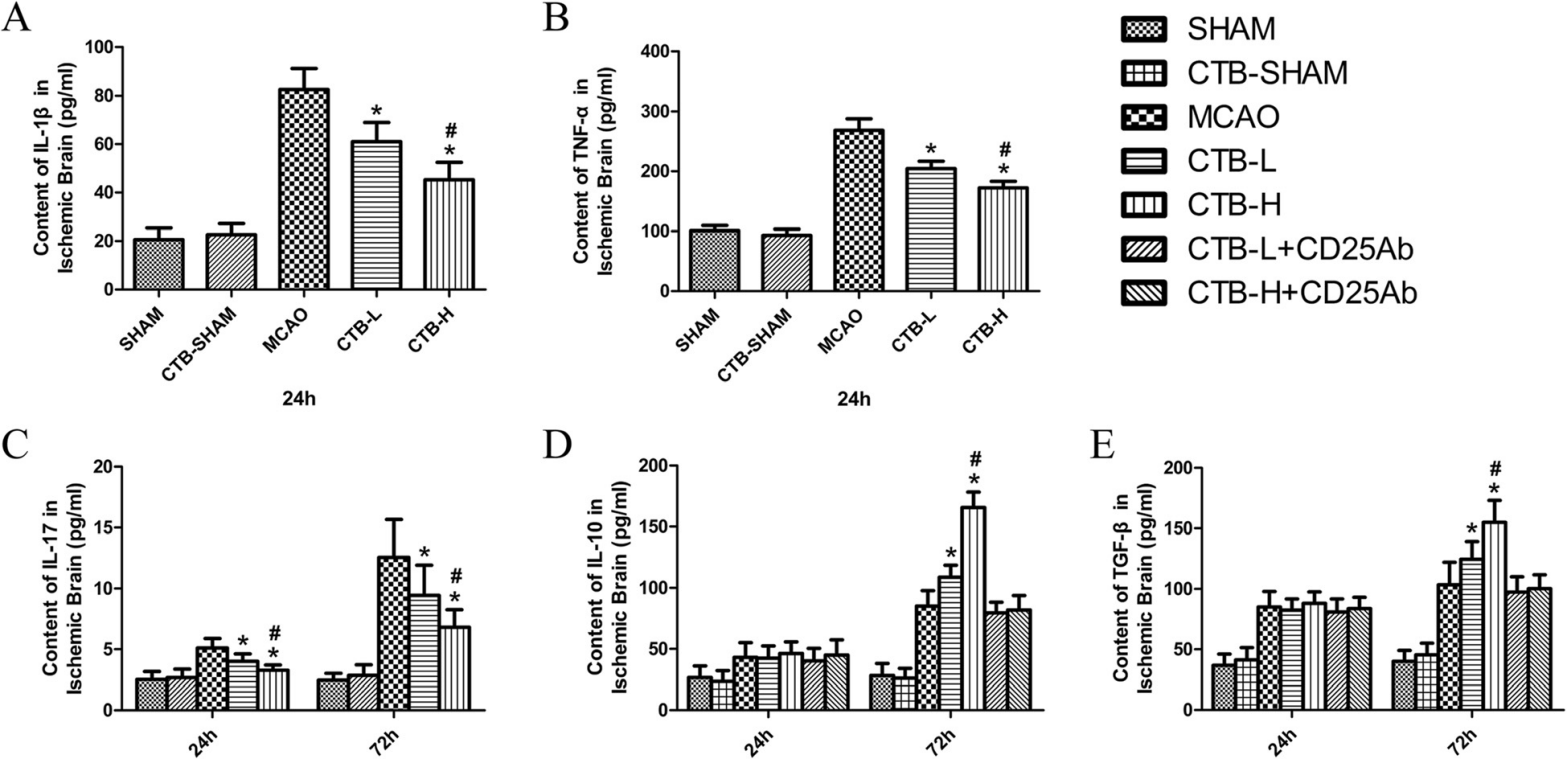
Effect of CTB on cytokine expression in the ischemic brain after MCAO. **a** The level of IL-1β in the sham ischemic hemisphere of the CTB-sham group was not significantly different from that of the sham group. CTB dose-dependently downregulated the levels of IL-1β in the ischemic brain at 24 h after ischemia. **b** The level of TNF-α in the sham ischemic hemisphere of the CTB-sham group was not significantly different from that of the sham group. CTB dose-dependently downregulated the levels of TNF-α in the ischemic brain at 24 h after ischemia. **c** The levels of IL-17 in the sham ischemic hemisphere of the CTB-sham group at 24 and 72 h after ischemia were not significantly different from those of the sham group at 24 and 72 h after ischemia, respectively. CTB dose-dependently downregulated the levels of IL-17 in the ischemic brain both at 24 and 72 h after ischemia. **d** The levels of IL-10 in the sham ischemic hemisphere of the CTB-sham group at 24 and 72 h after ischemia were not significantly different from those of the sham group at 24 and 72 h after ischemia, respectively. CTB did not affect the levels of IL-10 at 24 h after ischemia, while it dose-dependently upregulated the levels of IL-10 at 72 h after ischemia. Anti-CD25 pretreatment had no impact on the levels of IL-10 at 24 h, while it attenuated the up-regulations of IL-10 at 72 h after ischemia, both in the CTB-L group and CTB-H group. **e** The levels of TGF-β in the sham ischemic hemisphere of the CTB-sham group at 24 and 72 h after ischemia were not significantly different from those of the sham group at 24 and 72 h after ischemia, respectively. CTB did not affect the levels of TGF-β at 24 h after ischemia, while it dose-dependently upregulated the levels of TGF-β at 72 h after ischemia. Anti-CD25 pretreatment had no impact on the levels of TGF-β at 24 h, while it attenuated the up-regulations of TGF-β at 72 h after ischemia, both in the CTB-L group and CTB-H group. *Bars* represent mean ± SD (*n* = 8); *p < 0.05 vs MCAO group, and #*p* < 0.05 vs CTB-L group

To confirm whether CTB was immunomodulatory in experimental stroke, we tested the levels of several pro- and anti-inflammatory cytokines, including IL-1β, TNF-α, IL-17, TGF-β, and IL-10. The concentrations of IL-1β and TNF-α were measured by ELISA at 24 h after cerebral ischemia. The concentrations of IL-17, TGF-β, and IL-10 were measured by ELISA at 24 and 72 h after cerebral ischemia. In the ischemic brain, CTB treatment dose-dependently downregulated the levels of IL-1β, TNF-α, and IL-17, while the levels of TGF-β and IL-10 were unchanged at 24 h but dose-dependently upregulated at 72 h (Fig. 2).

**Fig. 3:**
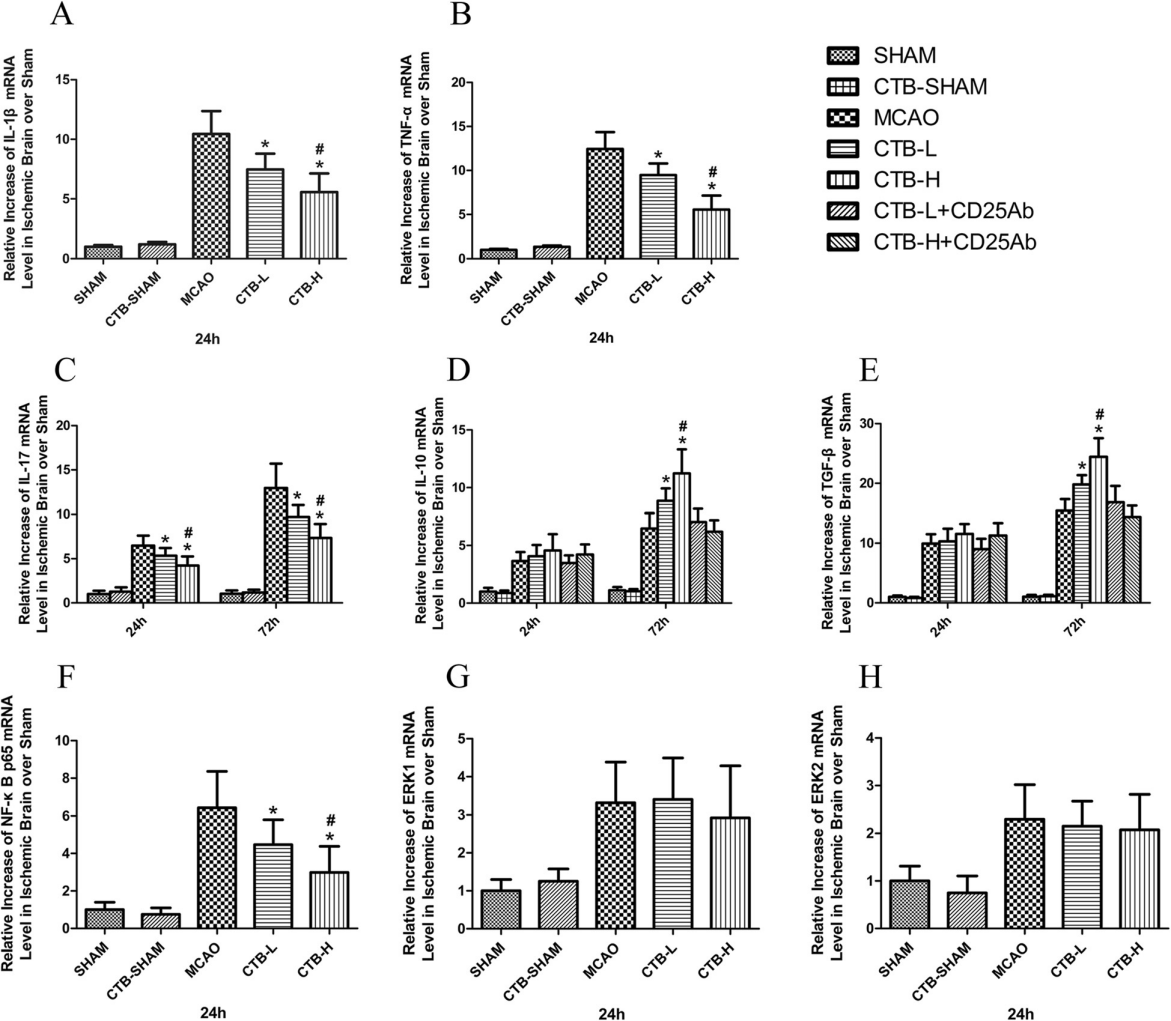
Effect of CTB on the mRNA expression of cytokines and transcriptional factors in the ischemic brain after MCAO. **a** The level of IL-1β mRNA expression in the sham ischemic hemisphere of the CTB-sham group was not significantly different from that of the sham group. CTB dose-dependently downregulated the level of IL-1β mRNA expression in the ischemic brain. **b** The level of TNF-α mRNA expression in the sham ischemic hemisphere of the CTB-sham group was not significantly different from that of the sham group. CTB dose-dependently downregulated the level of TNF-α mRNA expression in the ischemic brain. **c** The levels of IL-17 mRNA expression in the sham ischemic hemisphere of the CTB-sham group at 24 and 72 h after ischemia were not significantly different from those of the sham group at 24 and 72 h after ischemia, respectively. CTB dose-dependently downregulated the levels of IL-17 mRNA expression in the ischemic brain both at 24 and 72 h after ischemia. **d** The levels of IL-10 mRNA expression in the sham ischemic hemisphere of the CTB-sham group at 24 and 72 h after ischemia were not significantly different from those of the sham group at 24 and 72 h after ischemia, respectively. CTB did not affect the levels of IL-10 mRNA expression at 24 h after ischemia, while it dose-dependently upregulated the levels of IL-10 mRNA expression at 72 h after ischemia. Anti-CD25 pretreatment had no impact on the levels of IL-10 mRNA expression at 24 h, while it attenuated the up-regulations of IL-10 mRNA expression at 72 h after ischemia, both in the CTB-L group and CTB-H group. **e** The levels of TGF-β mRNA expression in the sham ischemic hemisphere of the CTB-sham group at 24 and 72 h after ischemia were not significantly different from those of the sham group at 24 and 72 h after ischemia, respectively. CTB did not affect the levels of TGF-β mRNA expression at 24 h after ischemia, while it dose-dependently upregulated the levels of TGF-β mRNA expression at 72 h after ischemia. Anti-CD25 pretreatment had no impact on the levels of TGF-β mRNA expression at 24 h, while it attenuated the up-regulations of TGF-β mRNA expression at 72 h after ischemia, both in the CTB-L group and CTB-H group. **f** The level of NF-kB p65 mRNA expression in the sham ischemic hemisphere of the CTB-sham group was not significantly different from that of the sham group. CTB dose-dependently downregulated the level of NF-kB p65 mRNA expression in the ischemic brain. **g** The level of ERK1 mRNA expression in the sham ischemic hemisphere of the CTB-sham group was not significantly different from that of the sham group. CTB did not affect the level of ERK1 mRNA expression in the ischemic brain. **h** The level of ERK2 mRNA expression in the sham ischemic hemisphere of the CTB-sham group was not significantly different from that of the sham group. CTB did not affect the level of ERK2 mRNA expression in the ischemic brain. *Bars* represent mean ± SD (*n* = 8); *p < 0.05 vs MCAO group, and #*p* < 0.05 vs CTB-L group

**Fig. 4:**
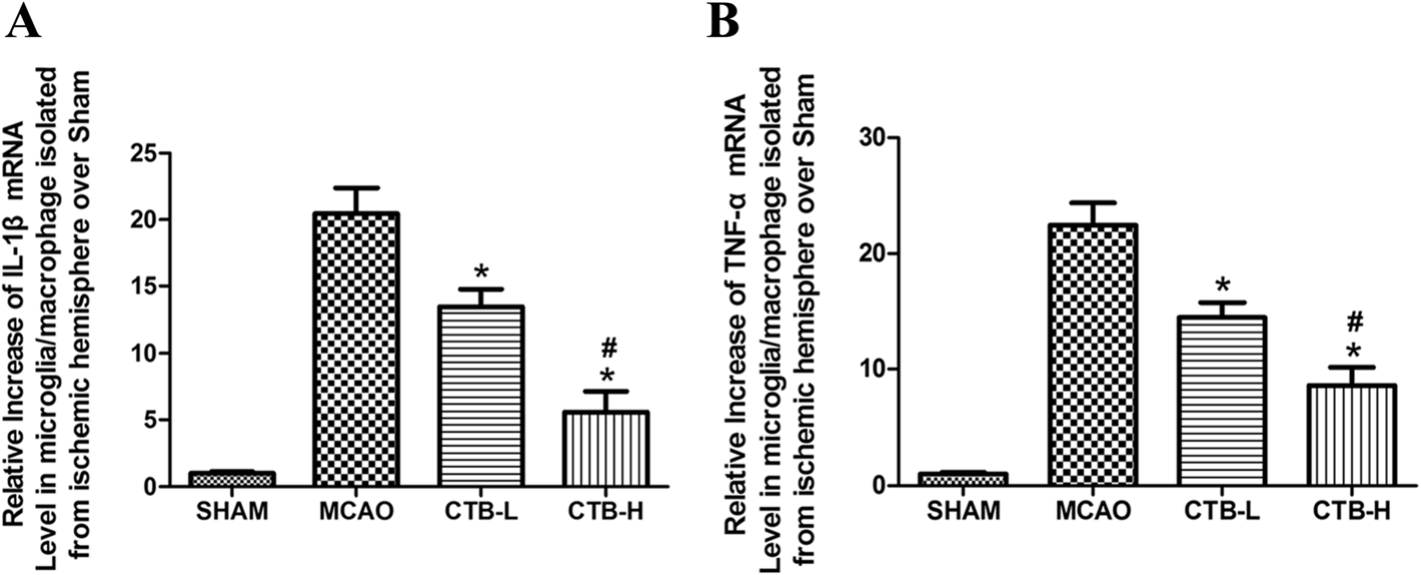
Effect of CTB on mRNA expression of cytokines in microglia/macrophage isolated from the ischemic hemisphere. **a** CTB dose-dependently downregulated the level of IL-1β mRNA expression in microglia/macrophage isolated from the ischemic hemisphere at 24 h. **b** CTB dose-dependently downregulated the level of TNF-α mRNA expression in microglia/macrophage isolated from the ischemic hemisphere at 24 h. *Bars* represent mean ± SD (*n* = 8); *p < 0.05 vs MCAO group, and #*p* < 0.05 vs CTB-L group

RT-PCR was used to assess the gene expressions in ischemic brain of IL-1β, TNF-α, IL-17, TGF-β, and IL-10 at the same time points as those in ELISA test. CTB treatment suppressed the gene expressions of IL-1β, TNF-α, and IL-17 (Fig. 3a–c), enhanced the gene expressions of TGF-β and IL-10 at 72 h (Fig. 3d, e), in a dose-dependent manner. Besides, CTB dose-dependently suppressed the mRNA expression of IL-1β and TNF-α in microglia/macrophage isolated from the ischemic hemisphere at 24 h (Fig. 4).

### Inactivation of Treg cells deprived the increase of TGF-β and IL-10 in the ischemic brain induced by CTB

To find out whether CTB increased the levels of TGF-β and IL-10 in the ischemic brain by induction of Treg cells, anti-CD25 pretreatment was conducted to inactivate Treg cells. Anti-CD25 pretreatment had no impact on the protein and mRNA levels of TGF-β and IL-10 at 24 h (Fig. 2d, e). The up-regulations of TGF-β and IL-10 at 72 h were attenuated by anti-CD25 pretreatment, both in CTB-L group and CTB-H group (Fig. 3d, e).

### CTB downregulated the expression of NF-kB p65 and binding activity of NF-kB, did not affect ERK1/2 expression, but suppressed ERK1/2 phosphorylation

**Fig. 5:**
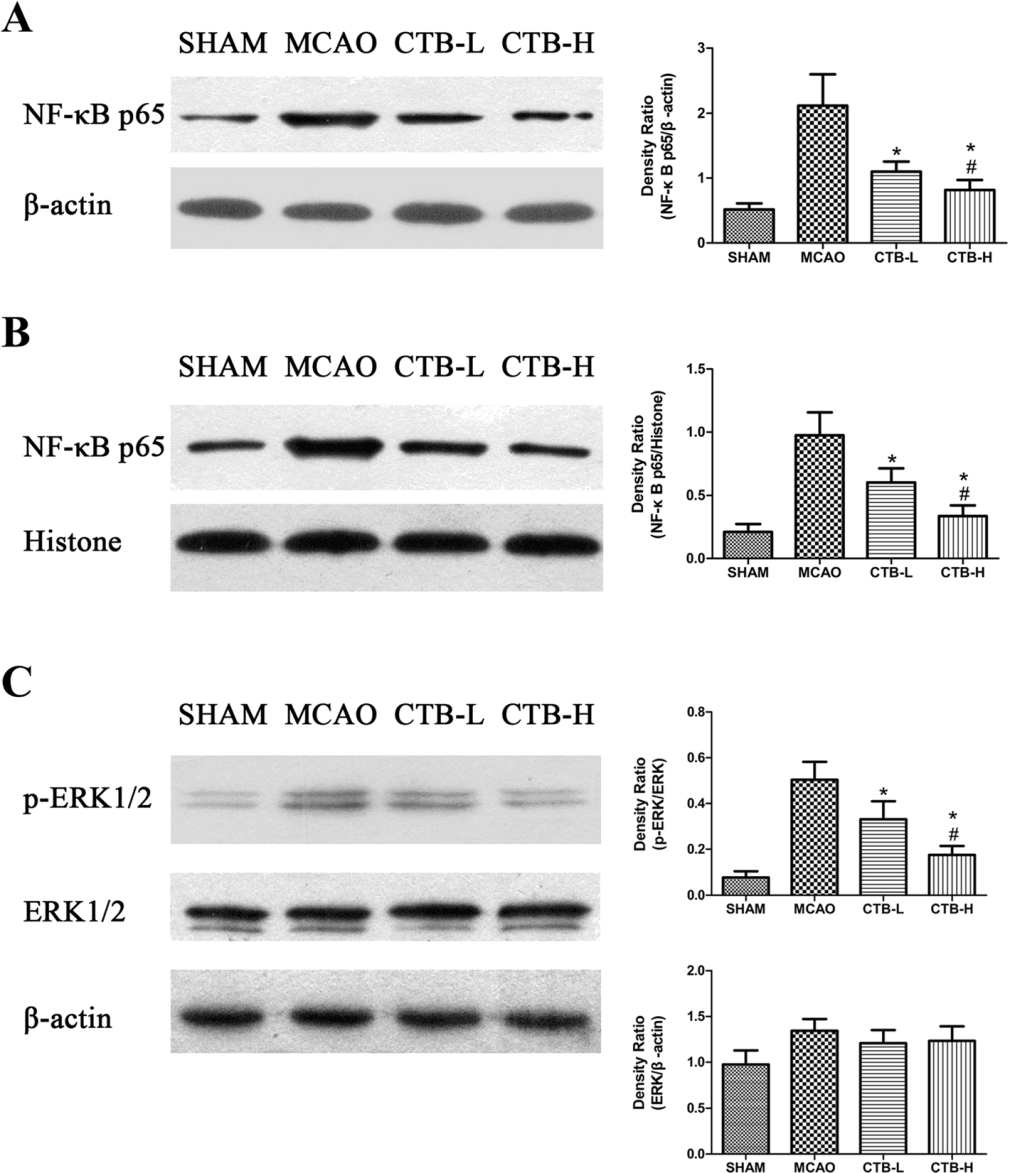
Effect of CTB on expression of cytosolic NF-kB p65, nuclear NF-kB p65, ERK1/2, and p-ERK1/2 in the ischemic brain after MCAO. **a** CTB dose-dependently downregulated the level of cytosolic NF-kB p65 expression in the ischemic brain. **b** CTB dose-dependently downregulated the level of nuclear NF-kB p65 expression in the ischemic brain. **c** CTB did not affect the level of ERK1/2 expression in the ischemic brain, but dose-dependently suppressed ERK1/2 phosphorylation. *Bars* represent mean ± SD (*n* = 8); *p < 0.05 vs MCAO group, and #*p* < 0.05 vs CTB-L group

To investigate whether CTB induced immunomodulation by affecting NF-kB p65 and ERK1/2 pathway, we measured the levels of NF-kB p65 (including cytosolic fraction and nuclear farction), ERK1/2, and p-ERK1/2 by Western blotting, the binding activity of NF-kB by EMSA, and the gene expression of NF-kB p65, ERK1, and ERK2 by RT-PCR in the ischemic brain at 22.5 h after reperfusion. The results of Western blotting showed that CTB treatment dose-dependently downregulated the levels of cytosolic NF-kB p65, nuclear NF-kB p65 in the ischemic brain (Fig. 5a, b). CTB treatment did not affect ERK1/2 expression, but dose-dependently suppressed ERK1/2 phosphorylation in the ischemic brain (Fig. 5c).

**Fig. 6:**
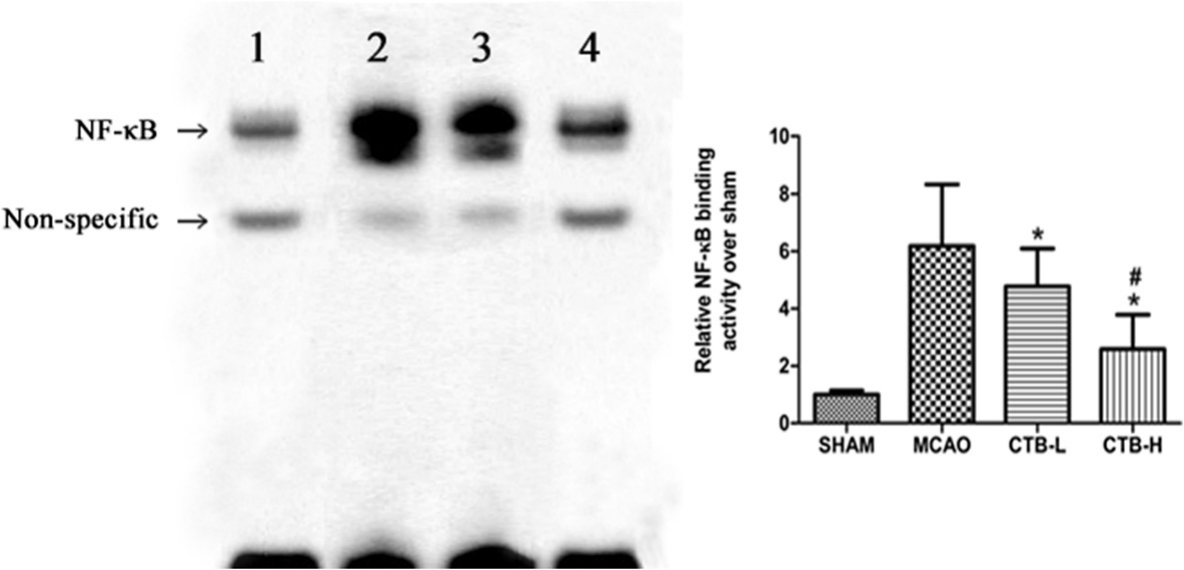
CTB treatment dose-dependently inhibited the binding activity of NF-kB in the ischemic hemisphere at 24 h after ischemia. Band *1*: SHAM; band *2*: MCAO; band *3*: CTB-L; band *4*: CTB-H. *Bars* represent mean ± SD (*n* = 8); *p < 0.05 vs MCAO group, and #*p* < 0.05 vs CTB-L group

The results of EMSA showed that CTB treatment dose-dependently inhibited the binding activity of NF-kB in the ischemic brain (Fig. 6).

The results of RT-PCR indicated that CTB treatment dose-dependently suppressed the gene expression of NF-kB p65 in the ischemic brain (Fig. 3f), but did not affect the gene expression of ERK1 and ERK2 (Fig. 3g, h).

### CTB suppressed the activation of microglia/macrophage induced by cerebral ischemia

**Fig. 7:**
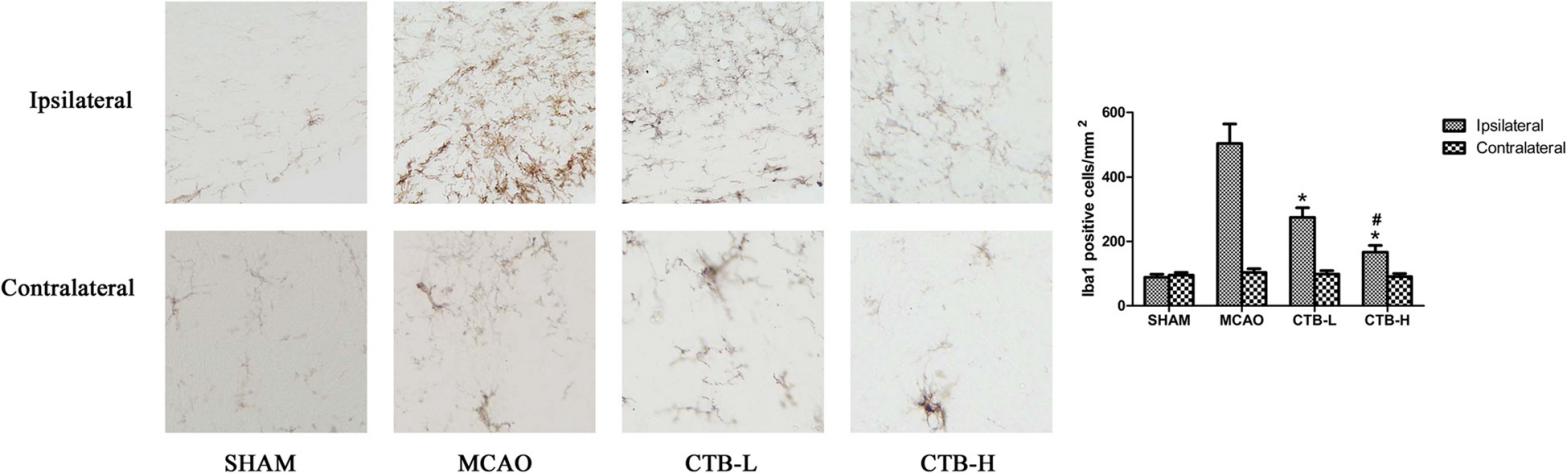
Immunohistochemistry for Iba1 in CTB dose-dependently suppressed microglia/macrophage activation at 24 h after ischemia in ischemic border. *Bars* represent mean ± SD (*n* = 8); *p < 0.05 vs MCAO group, and #*p* < 0.05 vs CTB-L group. Original magnification ×400

At 24 h after ischemia, the activation of microglia/macrophage stained by Iba1 around the infarct border in the MCAO group was markedly enhanced compared with that in the control group (Fig. 7). Treatment with CTB dose-dependently suppressed the expression of Iba1 (Fig. 7).

### Effects of CTB on the dynamic changes of Treg cells, γδT cells, IL-17-producing γδT cells, and Th17 cells

**Fig. 8:**
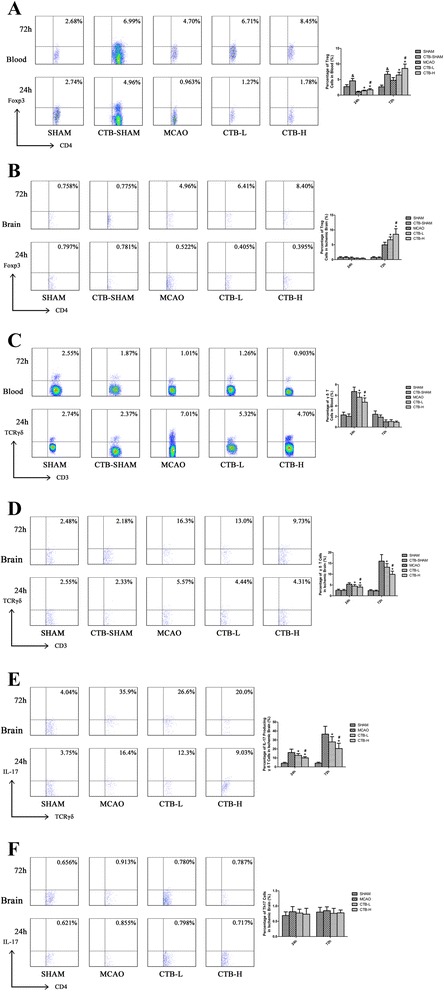
Effect of CTB on Treg cells, γδT cells, IL-17-producing γδT cells, and Th17 cells. **a** Treg cells in the peripheral blood of the CTB-sham group were significantly increased both at 24 and 72 h after ischemia, compared with those of the sham group. CTB dose-dependently increased Treg cells in the peripheral blood both at 24 and 72 h after ischemia. **b** Treg cells in the sham ischemic hemisphere of the CTB-sham group both at 24 and 72 h after ischemia were not significantly different from those in the sham ischemic hemisphere of the sham group. In the ischemic hemisphere, CTB did not alter the percentage of Treg cells at 24 h, but a dose-dependent increase was found at 72 h after ischemia. **c** γδT cells in the peripheral blood of CTB-sham group both at 24 and 72 h after ischemia were not significantly different from those of the sham group. CTB dose-dependently suppressed the increase of γδT cells at 24 h, while it did not affect the proportion of γδT cells at 72 h after ischemia in the peripheral blood. **d** γδT cells in the sham ischemic hemisphere of the CTB-sham group both at 24 and 72 h after ischemia were not significantly different from those in the sham ischemic hemisphere of the sham group. In the ischemic hemisphere, the increases of γδT cells at 24 and 72 h after ischemia were dose-dependently suppressed by CTB. **e** In the ischemic hemisphere, the increases of IL-17-producing γδT cells at 24 and 72 h after ischemia were dose-dependently suppressed by CTB. **f** In the ischemic hemisphere, CTB had no effect on the Th17 cells at 24 and 72 h after ischemia. *Bars* represent mean ± SD (*n* = 8); *p < 0.05 vs MCAO group, #*p* < 0.05 vs CTB-L group, and &*p* < 0.05 vs SHAM group

In the peripheral blood, Treg cells were decreased at 24 h and increased at 72 h after cerebral ischemia (Fig. 8a). In the ischemic hemisphere, no change of Treg cells was found at 24 h and an increase was detected at 72 h after cerebral ischemia (Fig. 8b). CTB dose-dependently compensated the decrease at 24 h and further increased Treg cells at 72 h in the peripheral blood after cerebral ischemia (Fig. 8a). In the ischemic hemisphere, CTB did not alter the percentage of Treg cells at 24 h, but dose-dependently increased Treg cells at 72 h after cerebral ischemia (Fig. 8b).

In contrast with Treg cells, γδT cells were increased in peripheral blood at 24 h after cerebral ischemia, while they were decreased to a level lower than that of sham group at 72 h (Fig. 8c). In the ischemic hemisphere, γδT cells increased at 24 h and further at 72 h after cerebral ischemia (Fig. 8d). CTB dose-dependently suppressed the increase of γδT cells in peripheral blood at 24 h after cerebral ischemia, while it had no effect on the decrease of γδT cells at 72 h (Fig. 8c). In the ischemic hemisphere, the increases of γδT cells at 24 h and 72 h after cerebral ischemia were suppressed by CTB in a dose-dependent manner (Fig. 8d). To confirm the source of IL-17 in the ischemic hemisphere, IL-17-producing γδT cells and Th17 cells were tested. The dynamic changes of IL-17-producing γδT cells in the ischemic hemisphere with or without CTB treatment were corresponding to those of γδT cells (Fig. 8e). In the ischemic hemisphere, no change of Th17 cells was found at 24 h and 72 h, and CTB treatment had no effect on Th17 cells (Fig. 8f).

### Effects of CTB on baseline values of cytokines, transcription factors, and peripheral cells

Considering the immune nature of CTB, the CTB-sham group was set up to evaluate the effects of CTB on baseline values of cytokines and peripheral cells. In brain, CTB did not affect the levels of IL-1β, TNF-α, IL-17, TGF-β, IL-10, NF-kB p65, ERK1, and ERK2 (Figs. 2 and 3). Besides, CTB had no effect on Treg cells and γδT cells in the brain (Fig. 8b, d). In the peripheral blood, CTB increased Treg cells at both 24 and 72 h (Fig. 8a), while it did not affect γδT cells (Fig. 8c).

Immunity plays an important role in the pathogenesis of ischemic stroke. The immune-inflammatory response in stroke begins within the brain and its vessels. The first immune cells that respond to these events seem to be brain-intrinsic microglia, followed by leukocytes and lymphocytes that enter the brain from the periphery. The proteins that these cells produce have an important role in cell death and in the enlargement of brain lesions that result from stroke [18]. The immune response can also contribute to pathogenesis before the onset of stroke: aberrant immune responses can induce inflammation within and around vessel walls, thereby promoting thrombosis, altering vascular reactivity, and encouraging atherosclerosis [19]. Leukocytes contribute to the growth of atherosclerotic plaques, leading to inflammation, instability, and rupture, and occlusion of arteries by atherosclerotic plaques leads to ischemic events [20]. Several controlled trials have assessed the efficacy of various immunomodulatory drugs in patients with ischemic stroke, such as anti-ICAM-1 antibody [21], minocycline [22], and fingolimod [23]. On the other hand, stroke has a marked impact on the immune system. Within days of stroke, patients develop lymphopenia [24]. The volume of the cerebral infarction has been directly associated with the extent of lymphocytopenia and monocyte dysfunction [25]. The down-regulation of immune responses that originates in the injured brain avoids autoimmunity against brain antigens that are released during cell death. However, post-stroke immunosuppression raise the incidence of infection which is associated with the increase of mortality [26].

CTB is a potent immunomodulator and has been studied as a therapy for autoimmune diseases in animal models, such as experimental autoimmune encephalomyelitis and diabetes [7, 9]. However, little is known about whether CTB is beneficial for stroke. In the present study, we clearly demonstrated that CTB protected the brain against ischemia and reperfusion injury in a rat MCAO model. CTB significantly ameliorated neurological deficits and cerebral infarct volume with a dose-effect relation. This protection was correlative with the anti-inflammation effect of CTB.

Increased expression cytokines play an important role in inflammatory response followed cerebral ischemia. Ischemia leads to rapid up-regulation of pro-inflammatory cytokines, such as TNF-α and IL-1β [14, 27]. In mice, intracerebroventricular injection of TNF-α or IL-1β enhances brain ischaemic damage [28, 29]. Researchers also found that the injection of antagonists of TNF-α and IL-1β relieved the ischemia injury [28, 30]. Experimental data have shown that resident microglia are activated within minutes of ischemia onset and produce TNF-α and IL-1β [31, 32], subsequent infiltrating macrophage also contribute the production of TNF-α and IL-1β. Inhibiting microglia/macrophage activation has been shown to protect brain against focal cerebral ischemia [14]. Our study showed that CTB treatment markedly suppressed the elevation of TNF-α and IL-1β in both ischemic brain and the microglia/macrophage isolated from the ischemic hemisphere, and microglia/macrophage activation was also inhibited. So one of the neuroprotective mechanisms of CTB might be attributed to the decrease of TNF-α and IL-1β via inhibiting microglia/macrophage activation.

IL-17 is another powerful pro-inflammatory cytokine. Th17 cells and γδT cells are considered as two major sources of IL-17. Experimental evidence demonstrated that the main source of IL-17 in the ischemic brain was γδT cells which could reach the damaged cerebral hemisphere as early as 6–12 h after ischemia and peaked at 3 days [33]. Our results also showed that the dynamic change of IL-17 in the ischemic brain was paralleled with that of γδT cells and IL-17-producing γδT cells; meanwhile, no change of Th17 cells was detected, suggesting that CTB reduced IL-17 by suppressing γδT cells. CTB suppressed the increase of γδT cells in the peripheral blood at 24 h after ischemia, indicating that CTB might prevent the release of γδT cells from immune organs to blood so less γδT cells could infiltrate into the brain. Previous studies have demonstrated that blockade of T cell infiltration into the brain reduced cerebral ischemic damage [13, 33], indicating that γδT cells deteriorate ischemic stroke by migrating to the brain instead of signaling from outside of the brain. Therefore, we proposed that CTB improved stroke by inhibiting the infiltration of γδT cells into the brain instead of suppressing the increase of γδT cells in the peripheral blood.

The outcome of post-ischemic inflammatory processes mainly depends on the imbalance between the activation of pro-inflammatory cytokine cascade and the induction of anti-inflammatory cytokines [34]. IL-10 and TGF-β are two major anti-inflammatory cytokines and could ameliorate experimental stroke [35]. It has been demonstrated that Treg cells reached the ischemic brain at several days after ischemia and exerted protective effect by secreting IL-10 and TGF-β [13]. In our study, the dynamic changes of IL-10 and TGF-β in the ischemic brain were consistent with that of Treg cells. So CTB might increase IL-10 and TGF-β in the ischemic brain by inducing more Treg cells migrating into the infarct area. Inactivation of Treg cells attenuated the raise of IL-10, and TGF-β also indicated that the major source of these cytokine increments was Treg cells. Besides, since CTB could induce the production of Treg cells, the increase of Treg cells in the peripheral blood might also contribute to the increase of Treg cells infiltrating into the ischemic brain. Although CTB had no effect on γδT cells at 72 h in the peripheral blood after cerebral ischemia, there is still the risk of enhancing the post-stroke immunodeficiency since Treg cells were increased. It has been demonstrated that TGF-β is induced in response to focal cerebral ischemia [36]. Different brain cells, including neurons, astrocytes, and microglias, can secrete TGF-β after MCAO [37]. Our results also showed that the level of TGF-β in the ischemic brain tissue of the MCAO group was higher than that of the sham group. The up-regulation of TGF-β could last more than 1 week after stroke [38]. Many researches have shown the neuroprotective effect of TGF-β in stroke; however, increment of TGF-β during chronic injury leads to reactive astrogliosis. Astrogliosis may have a detrimental effect on recovery due to astrocytes extending processes in the infarct border to encompass the developing infarct, thereby inhibiting neuronal plasticity and the formation of new axons and blood vessels in the infarcted region [39]. Therefore, it seems that a short-term usage of CTB might be reasonable.

NF-kB, a pivotal transcription factor, is essential for immune and stress responses within the brain. It is composed of subunits p65 and p50. When cells are stimulated, NF-kB is activated and translocated into the nucleus, inducing transcriptional activation of potentially deleterious pro-inflammatory genes, such as TNF-α and IL-1β [40]. In rodents, activation of NF-kB occurs after experimental stroke, and inhibiting the NF-kB signaling pathway by pharmacological and genetic approaches has been reported to be neuroprotective in the model of experimental stroke [40, 41]. One mechanism responsible for NF-kB induction involves the stimulation of the mitogen-activated protein kinase (MAPK) pathways. Three members of the MAPK family, ERK1/2, SAPK/JNK, and p38, are all activated early after focal cerebral ischemia, and inhibition of this pathway reduces the expression of proinflammatory cytokines and confers protection against the insult [42]. In this study, we found that CTB treatment did not affect ERK1/2 expression, but suppressed ERK1/2 phosphorylation. NF-kB expression was downregulated, and its translocation into the nucleus was also suppressed. Besides, the binding activity of NF-kB was inhibited. Therefore, we hypothesized that CTB affects the NF-kB pathway by inhibiting ERK1/2 phosphorylation, although we could not exclude that SAPK/JNK and p38 were simultaneously involved. It was reported that neuronal NF-kB inhibition reduced stroke size and apoptosis [10]. It was also shown that over-expression of NF-kB-p65 in astrocytes significantly increased the co-cultured neuronal apoptosis under oxygen-glucose deprivation followed by oxygen-glucose regeneration condition [31]. In addition, suppression of NF-kB in microglia attenuated the severity of experimental stroke [32]. So it is unclear that CTB downregulated NF-kB in which cell types.

The increment of peripheral Treg cells in the CTB-sham group reflected the immune tolerance induced by CTB. Since the blood-brain barrier was not damaged, Treg cells in the brain did not increase. On the other side, the reason for γδT cells being not suppressed might be that these cells were not in activating state in the CTB-sham group. No changes of the levels of cytokines and transcription factors in the brain of CTB-sham group were found, indicating that CTB might only have impact on the brain with inflammation.

Finally, we have concerns about the pros and cons of using CTB as a potential clinical therapy to stroke. CTB did ameliorate ischemic stroke by immunomodulation. However, it should be noted that stroke per se could lead to immunosupression which promotes spontaneous bacterial infections [43]. And infection is an independent risk factor for poor outcome after acute ischemic stroke [44]. So it is possible that CTB might increase infection after stroke by enhancing immunodeficiency, and this impact might be attenuated by the specific pathogen-free environment in which our study was done. Therefore, further research is required to evaluate the risk of raising infection after stroke by CTB.

## Conclusions

Administration of CTB decreased neurologic impairment and tissue injury after cerebral ischemia via its immunomodulatory functions, including inhibiting microglia/macrophage activation, suppressing γδT cells, and inducing production of Treg cells, thus regulating the secretion of related cytokines. Suppression of NF-kB and ERK1/2 pathways is involved in the neuroprotective mechanism of CTB.

## Abbreviations

CTB, cholera toxin B subunit; MAPK, mitogen-activated protein kinase; MCAO, middle cerebral artery occlusion; PBMC, peripheral mononuclear blood cells; Treg, regulatory T
